# Feeding Behavior of Asian Citrus Psyllid [*Diaphorina citri* (Hemiptera: Liviidae)] Nymphs and Adults on Common Weeds Occurring in Cultivated Citrus Described Using Electrical Penetration Graph Recordings

**DOI:** 10.3390/insects11010048

**Published:** 2020-01-10

**Authors:** Justin George, Ramdas Kanissery, El-Desouky Ammar, Itze Cabral, Larry T. Markle, Joseph M. Patt, Lukasz L. Stelinski

**Affiliations:** 1 Department of Entomology and Nematology, Citrus Research and Education Center, University of Florida, 700 Experiment Station Rd., Lake Alfred, FL 33850, USA; justin.george@usda.gov (J.G.); Eldesouky.ammar@ars.usda.gov (E.-D.A.); cabralitze@ufl.edu (I.C.); 2 United States Department of Agriculture, Agricultural Research Service, 2001 South Rock Road, Fort Pierce, FL 34945, USA; larry.markle@usda.gov (L.T.M.); joseph.patt@usda.gov (J.M.P.); 3 Department of Horticulture Sciences, Southwest Florida Research and Education Center, University of Florida, 2685 SR 29 North Immokalee, Immokalee, FL 34142, USA; rkanissery@ufl.edu

**Keywords:** Asian citrus psyllid, Huanglongbing, electrical penetration graph, weed species, xylem feeding

## Abstract

Asian citrus psyllid, *Diaphorina citri*, transmits *Candidatus* Liberibacter asiaticus (*C*Las), the putative causal agent of Huanglongbing disease. Although they primarily feed on the phloem of *Citrus* and related plants, when grove or host conditions are unfavorable, *D*. *citri* may be able to use weed species as alternate food sources for survival. To explore this possibility, electrical penetration graph (EPG) recordings (18 h) were performed to investigate the feeding behavior of psyllid adults and nymphs on three common south Florida weeds (*Bidens alba*, *Eupatorium capillifolium*, and *Ludwigia octovalvis*). EPG recordings revealed that the proportion of time spent by *D. citri* feeding on xylem was similar on all tested weed species (19%–22%) and on the positive control (20%), the preferred host, *Citrus macrophylla*. Very little to no phloem feeding was observed on weed species by either nymphs or adults. Histological studies using epifluorescence microscopy showed that salivary sheaths were branched and extended into xylem of weed species, whereas they ended in phloem on citrus plants. No choice behavioral assays showed that adults can obtain some nutrition by feeding on weed species (xylem feeding) and they may be able to survive on them for short intervals, when host conditions are unfavorable.

## 1. Introduction

Citrus greening, aka Huanglongbing (HLB), is the most destructive citrus disease in the world; it is presumably caused by *Candidatus* Liberibacter asiaticus [*C*Las], a phloem-inhabiting bacterium [1]. *C*Las is transmitted by Asian citrus psyllid, *Diaphorina citri*, a tiny hemipteran which reproduces only on the young shoots of *Citrus* and a few closely related genera [2,3]. An important component of HLB management in citrus is management of the vector, *D. citri* [4,5,6]. According to a U.S National Academy of Sciences report, there is an imperative need for *D*. *citri* control to prevent repeated inoculum transfer and reduce the spread of HLB [7]. Insecticide applications and abiotic stressors can induce psyllids to disperse from groves [8,9,10]. Under these circumstances, *D. citri* may utilize secondary host plants, such as the weeds that are abundant in and around Florida citrus groves, as temporary hosts for facilitating survival [8]. To optimize area-wide management of *D*. *citri*, it is therefore essential to determine the psyllid’s ability to feed and survive on potential weed hosts that are abundant in and around citrus groves.

Previous studies by Johnston et al. [10] showed that *D. citri* may be able to use weeds as a ‘way station’ where shelter and perhaps other resources are obtained during its search for its preferred host, *Citrus*. The three weed species investigated were Spanish needle [*Bidens alba* (L.)], dogfennel [*Eupatorium capillifolium* (Lam.)], and primrose-willow [*Ludwigia octovalvis* (Jacqu.)]. In Florida, these weed species grow in close proximity to citrus trees and can affect tree growth and influence pest and natural enemy populations. Weeds growing near tree trunks may also create a favorable environment for pathogens that may infect the trunk and roots [11]. Weed species that are prevalent in the ditches and beds of poorly managed citrus groves could act as secondary or alternate host plants for psyllids following insecticide sprays or during dispersal [3].

Spanish needle (*B. alba*), also known as common beggar’s tick, is a perennial, herbaceous weed frequently found in citrus groves. It belongs to the Asteraceae family and is commonly found throughout the southeastern United States [12]. Spanish needles can dominate the grove understory, and consequently compete with citrus for resources and interfere with grove operations. If left unmanaged, Spanish needles can reduce citrus growth, particularly affecting younger trees [13,14]. While this weed can be managed chemically by pre- and post-emergent herbicides, its ability for massive seed production in combination with fast re-establishment from soil seed banks results in a year-round presence [15]. Dogfennel (*E. capillifolium*) is another perennial weed from the Asteraceae that is found throughout the Southeast United States. It is troublesome in cultivated citrus and is the most common pasture weed in Florida [16]. The plant is characterized by succulent, thick and furry stems and lace-like foliage, and grows 1.8 m or more in height. Infestations of dogfennel are dense and management in the infested areas is challenging. Mexican primrose-willow (*L. octovalvis*) is a weed that also thrives in cultivated citrus, especially in drainage ditches and banks characteristic of modern grove architecture. A member of Onagraceae, it is highly tolerant to fluctuating water levels becoming abundant near irrigation canals and drainage ditches [17]. The weed is resistant to harsh conditions; for instance, their stem tissues swell rapidly when immersed in water as a defense mechanism against waterlogging [18].

In laboratory assays, Johnston et al. [10] showed that survival of *D. citri* adults on these three weed species was approximately two-fold greater (48–96 h) than under starvation conditions with water alone. Choice assays showed no differences in the host selection between *Citrus* plants and these weed species for up to 72 h [10]. Detailed feeding assays were lacking in this reported study and feeding behaviors of psyllids on these weed species remained unknown. Determining psyllid feeding behavior, particularly xylem and phloem feeding activities on weed species, is necessary to understand the dynamics of their movement, survival, and persistence in and around citrus groves.

Electrical penetration graph (EPG) technology is an optimal tool for quantification of cryptic feeding behaviors of hemipterans as they occur within leaf tissues [19]. EPGs are recordings of resistance changes (electrical potential) following stylet penetration into specific plant tissues and allow quantification of the amount of time spent in feeding and/or penetration activities [19]. In this study, we used EPG recordings to understand the feeding behavior of *D. citri* nymphs and adults on Spanish needle, dogfennel, and Mexican primrose. These were compared to EPG recordings made on a true host of *D. citri*, *Citrus macrophylla* Wester, also called alemow. We hypothesized that ACP utilizes weeds as a secondary host plant resource when preferred hosts are unavailable. However, the potential nutritional value or mechanisms by which such secondary hosts could affect *D. citri* behavior were both unknown. The weeds species investigated, Spanish needle, dogfennel, and Mexican primrose willow, were selected based on their persistent occurrence in citrus groves throughout Florida [20] and previous reports implicating them as secondary hosts [10]. No choice behavioral assays were performed using *D. citri* nymphs and adults to investigate survival and development on weed species in comparison with true hosts. Epifluorescence microscopy of leaf tissues and salivary sheaths were performed to investigate position of salivary sheaths in vascular tissues. The main objective of this study was to describe the feeding behavior of *D. citri* on weed species, and understand their potential contribution to spread of HLB.

## 2. Materials and Methods

### 2.1. Insects

Adult *D. citri* were obtained from a colony established in 2000 at the USDA-ARS U.S. Horticultural Research Laboratory, Fort Pierce, FL. The psyllids were originally collected from citrus in the field and subsequently reared in greenhouse cages containing orange jasmine, *Murraya exotica* L. (*M. paniculata* auct. non.), and more recently *C. macrophylla* Wester as described by Skelley and Hoy [21]. The colony was tested quarterly by qPCR according to Li et al. [22] to confirm absence of *C*Las. All nymphs used for EPG recordings were early 4th instars; all adults used were 7–10 days old. It has been established that no differences exist between EPG waveforms produced by male and female *D. citri* [23,24]. Therefore, the sex of the psyllids used was not considered as a factor.

### 2.2. Plants

Weed species evaluated in behavioral assays and electrical penetration graph recordings were Spanish needle, dogfennel, and Mexican primrose-willow. Weed plants were collected from established natural locations at an early stage of growth and transplanted individually into 3.8-L plastic pots filled with a 2:1 potting mix: sand mixture (Pro-Mix BX Mycorrhizae growing medium, Quakertown, PA, USA). Plants were grown in a temperature-controlled greenhouse under natural sunlight conditions. *C. macrophylla*, a known host of *D. citri*, was used as a positive control and maintained under identical conditions. Young leaves (soft, immature, and fully expanded) of *C. macrophylla* and weed species were used in the behavioral assays and EPG recordings.

### 2.3. Electrical Penetration Graph Recordings of D. citri Nymphs and Adults

Plants were washed and watered 24 h prior to the EPG experiment. Young, fully expanded leaves were selected for EPG recordings with *D. citri* and for subsequent histological examination using fluorescence microscopy. EPG recordings were conducted with a DC-monitor (GIGA-8 model, EPG-Systems, Wageningen, The Netherlands) adjusted to 50× gain [19]. The analog signal was digitized through a DI-710 board and displayed using Windaq Lite ver. 2.40 software (Dataq Instruments Inc. Akron, OH, USA) on a Dell desktop computer. The EPG monitoring system was housed in a grounded Faraday cage in an environmentally controlled room under continuous photophase. The temperature was set to 26 °C with 60%–65% RH. Psyllid nymphs or adults were aspirated into glass vials and starved for 2 h prior to the start of the experiment. Psyllids were then placed in a freezer (−4 °C) for 45–60 s to immobilize them and held by a plastic pipette tip connected to a gentle vacuum supply under a dissecting microscope. Each psyllid was attached to a 25 µm-diam. gold wire (Sigmund Cohn Corp., Mt. Vernon, New York, NY, USA) by a droplet of silver conducting paint (Ladd Research Industries, Burlington, VT, USA) applied to the pronotum [19,24]. The gold wire lead was attached to a copper electrode (3 cm × 1 mm diameter) connected to the EPG probe. To complete the electrical circuit, a reference copper electrode (10 cm × 2 mm) was inserted into the soil medium near the base of each plant. Psyllid nymphs and adults were restricted to the abaxial surface of fully expanded leaves of weeds or citrus, the preferred feeding site for nymphs [24,25,26,27]. The feeding behaviors of individual *D. citri* adults and nymphs were monitored on Spanish needle (n = 22), Mexican primrose (n = 18), Dogfennel (n = 18), and *C. macrophylla* (n = 22) for a continuous period of 18 h.

Characterization of EPG waveforms was accomplished by visually identifying and annotating waveforms based on comparison to prior histological studies [24,27]. Windows Dataq waveform browser (Dataq Instruments Inc., Akron, OH, USA) was used to annotate waveforms. The number and duration of waveform bouts were tabulated in an electronic spreadsheet. The waveforms were visually inspected for frequency patterns and annotated as non-probing (Np), mesophyll intercellular pathway (C), phloem penetration (D), phloem salivation (E1), phloem ingestion (E2), or xylem ingestion (G) phases. Statistical analysis was performed using JMP (v. 10, SAS Inc, Cary, NC, USA). A 2 × 4 factorial design was used to evaluate main effects and interaction of psyllid stage (nymph or adult) and plant species (Spanish needle, Mexican primrose, dogfennel, and citrus) on the number of bouts corresponding to described waveforms and the total duration of each waveform during each 18 h of EPG recording.

### 2.4. Visualization of Salivary Sheaths Produced by D. citri Adults

Ten adult psyllids were fed on the abaxial side of the leaves inside meshed clip cages for four days (n = 3). One cm of the central midrib on each leaf was left uncovered for psyllids to feed. The rest of the leaf surface was covered with labelling tape. After 4 d, the uncovered section of leaf was harvested for subsequent leaf sectioning to visualize salivary sheaths. The abaxial side was chosen so that it would be comparable with leaves used in the EPG study. The midrib was chosen because of the difficulty in cross sectioning the smaller secondary veins. From each leaf, 2–3 small pieces of the midrib ca. 5 mm long were cut with a sharp razor blade, fixed overnight in 4% paraformaldehyde in phosphate buffered saline (PBS), then washed 3 times in PBST (PBS + 0.1% Triton X 100). Each leaf section was placed in a drop of PBS on a microscope slide and sectioned by hand using a sharp razor blade to the thinnest possible sections under a stereomicroscope (at 20× or higher). These sections, ca. 50–70 μm thick as determined by confocal microscopy [28], were gently transferred (without staining) into a drop of Fluoro-Gel mounting medium (Electron Microscopy Sciences, Hatfield, PA, USA) on another microscope slide. Auto fluorescence of salivary sheaths and surrounding leaf tissues [28] was examined under UV light using an epifluorescence inverted microscope (Olympus IX70, with 4× or 10× objectives) fitted with a camera and an imaging program (CellSens software, Olympus, Tokyo, Japan). The occurrence, branching and position where sheaths terminated (=termini) were recorded for each leaf section examined.

### 2.5. No Choice Assays to Study the Survival of D. citri Adults and Nymphs on Weed Plants

In order to measure survival of psyllids on different weed hosts, we conducted no choice assays using meshed clip cages. Ten adult psyllids were introduced on a single leaf inside mesh clip cages for 12 days. Ten psyllids were placed inside cages with moistened cotton rolls (as a water source) as a negative control to quantify survivorship of psyllids under starvation conditions. The treatments were identical to those tested in EPG recordings and there were six replicates per plant species and for the negative control. An identical assay was conducted separately using newly emerged fourth instar nymphs. For each experiment, the number of surviving psyllid adults or nymphs was counted 2, 6, and 12 d after caging on plant treatments. To assess the influence of plant species on *D. citri* survival, we conducted analysis of variance (ANOVA) followed by Tukey’s HSD for comparison of means using JMP (v. 10, SAS Inc., Cary, NC, USA).

## 3. Results

### 3.1. Electrical Penetration Graph Recordings of D. citri Nymphs and Adults

**Figure 1:**
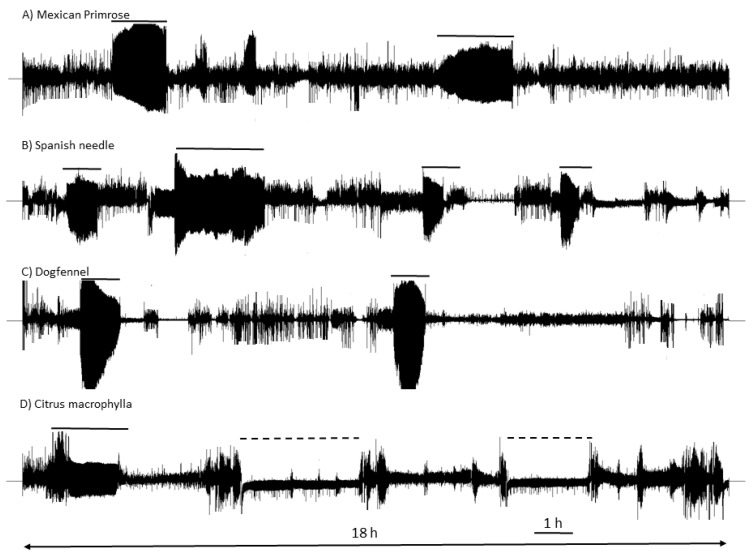
Example electrical penetration graph recordings illustrating feeding activities of *Diaphorina citri* adults on the weed species during 18 h recordings: (**A**) Mexican primrose, (**B**) Spanish needle, (**C**) Dogfennel, and a preferred host (**D**) *Citrus macrophylla*. Horizontal black lines indicate xylem feeding (G) and dashed black lines indicate phloem feeding (E2). Black horizontal scale bar shows 1 h.

Overall, the waveforms produced by both *D. citri* nymphs and adults during the 18 h recording periods were generally similar to those reported previously for them on citrus [25,27]. Examples of typical EPG recordings for adult psyllids on each of the four plant species tested are provided in Figure 1. The adults spent similar lengths of time engaged in xylem feeding activities on both weed species and *C. macrophylla* but only xylem feeding activities were observed on the weed species (Figure 1A–C). However, the adults engaged in both xylem and phloem-feeding activities on *C. macrophylla* (Figure 1D).

#### 3.1.1. Frequency of Phloem Feeding Activities is Higher in Citrus than in Weeds

Significant interactions were observed between psyllid life stage (nymph vs. adult) and plant type on the frequency (number of bouts) of waveforms corresponding to intercellular passage (C, aka stylet pathway), phloem penetration (D), phloem salivation (E1), phloem ingestion (E2) and non-probing (Np) activities (*p* < 0.01) (Table 1). Psyllid life stage had no effect on the frequency of xylem feeding (G) and intercellular stylet pathway (C) activities (Table 1). Nymphs performed more phloem feeding activities (D, E1, E2) on *C. macrophylla* plants than adult psyllids (Table 1, *p* < 0.0001). Nymphs performed some phloem penetration (D) and salivation (E1) attempts on weed species; however, no phloem feeding bouts (E2) were observed on them. In contrast, the nymphs engaged in frequent phloem feeding (E2) on *C. macrophylla* (*p* < 0.0001). Overall, fewer xylem feeding bouts were observed on *C. macrophylla* than on the weed species tested ((*p* = 0.002) Table 1). Periods of non-probing (Np) recorded by both nymphs and adults showed a similar pattern to that observed for intercellular stylet pathway (C). However, fewer non-probing activities were observed on dogfennel compared to primrose, Spanish needle, and *C. macrophylla* (Table 1, *p* = 0.01).

#### 3.1.2. Duration of Xylem Feeding Activities is Similar on Weed Species Compared to Citrus

**Figure 2:**
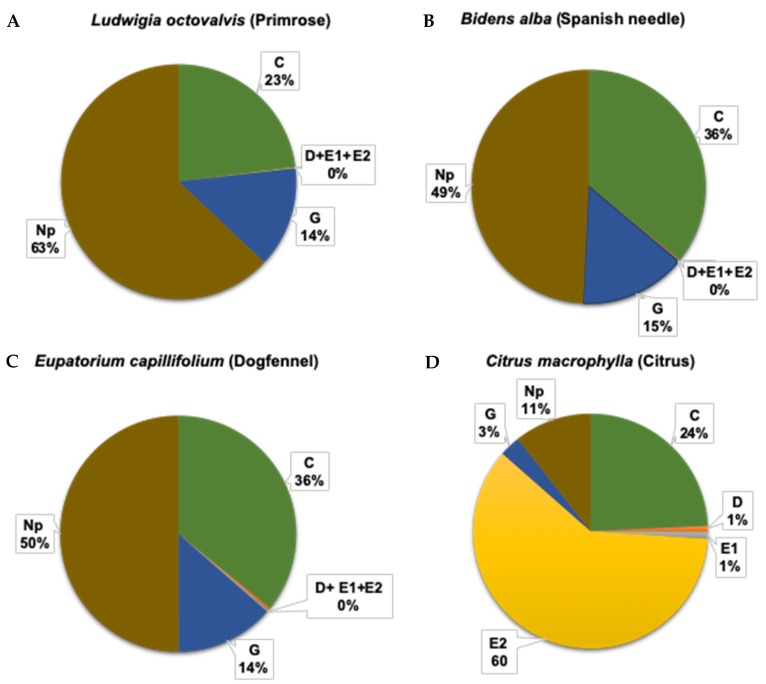
Percent duration of each feeding activity (C, D, E1, E2, G, and Np) performed by *Diaphorina citri* nymphs on various weed species and citrus during 18 h EPG recordings. (**A**) Primrose, n = 18; (**B**) Spanish needle, n = 22; (**C**) Dogfennel, n = 18; and, (**D**) *Citrus macrophylla*, n = 22.

**Figure 3:**
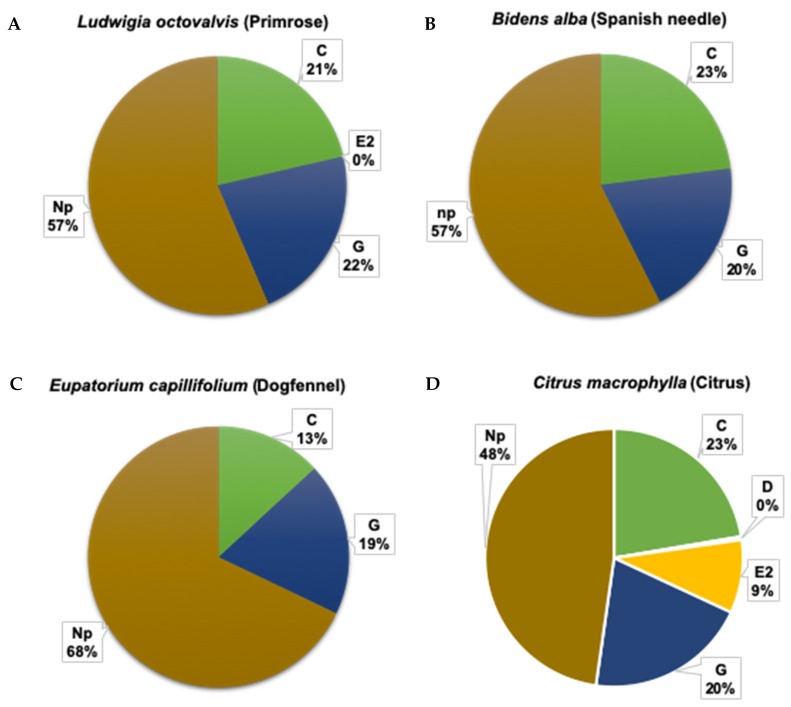
Percent duration of each feeding activity (C, D, E1, E2, G, and Np) performed by *Diaphorina citri* adults on various weed species and citrus during 18 h EPG recordings. (**A**) Primrose, n = 18; (**B**) Spanish needle, n = 22; (**C**) Dogfennel, n = 18; and, (**D**) *Citrus macrophylla*, n = 22.

There were no significant interactions between plant type and insect stage and no effect of plant type on the duration of the xylem feeding (Table 2). However, psyllid life stage had a significant effect on the mean duration of all waveforms recorded (Table 2, Figure 2 and Figure 3). The duration of individual bouts of waveforms C, D, E1 and E2 were significantly longer for nymphs than bouts conducted by adults (Table 2). The mean duration (± SEM) of bouts of phloem ingestion (E2) by nymphs (68 ± 10 min) was 5 times greater than that of adults (14 ± 10 min) (*p* = 0.004) (Figure 1, Figure 2 and Figure 3, Table 2). Phloem ingestion observed on *C. macrophylla* plants was significantly longer than on weed species (only three short phloem-feeding bouts totaling 20 min were observed during one EPG recording on primrose) (Table 2, *p* < 0.0001). The duration of phloem penetration and salivation (D, E1) was significantly longer for nymphs than adults. However, the mean duration (± SEM) of xylem ingestion bouts (G) was significantly longer (*p* = 0.004) for adults (39.5 ± 3.9 min) than nymphs (21.3 ± 4 min) (*p* = 0.004). No differences were observed for stylet pathway and xylem ingestion activities between the weed species and *C. macrophylla* (*p* = 0.10). In addition, adults (41 ± 3 min) spent significantly more time in non-probing (Np) activities than nymphs (30 ± 3 min) (*p* = 0.03, Table 2). The duration of non-probing activities was significantly shorter on *C. macrophylla* than on the various weed species, indicating that psyllids preferred citrus to weeds as a host.

#### 3.1.3. Total Duration (Frequency × Mean Duration) of Phloem and Xylem Feeding Activities

**Figure 4:**
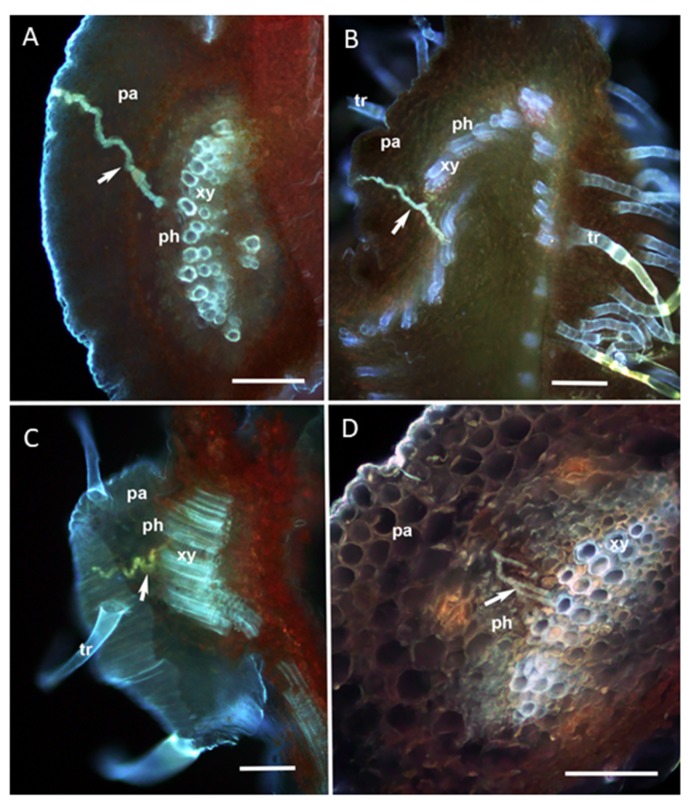
Fluorescence micrographs of cross section in the midribs of (**A**) *Citrus macrophylla*, (**B**) Dogfennel, (**C**) Primrose, and (**D**) Spanish needle leaves on which *Diaphorina citri* adults were caged for 4 days (n = 3). *D. citri* stylet tracks (salivary sheaths, arrows) terminated in the phloem (ph) in *C. macrophylla* (**A**), but terminated in xylem vessels (xy) in the three tested weeds (**B**–**D**). Abbreviations: pa: parenchyma; ph: phloem; tr: trichomes; xy: xylem. Scale bars = 50 µm.

Nymphs spent a significantly longer duration in stylet pathway activities (C) than adults (*p* < 0.0001). The total duration of stylet pathway activities was longer on Spanish needle than on the other plant species tested (*p* = 0.02). A significant interaction was observed between psyllid life stage and plant type on the total duration of C, E1, E2, and Np waveforms (Table 3). Total duration of phloem penetration (D), salivation (E1) and ingestion (E2) activities was significantly longer on *C. macrophylla* than on the weed species. Nymphs spent significantly more time in phloem penetration activities on *C. macrophylla* plants (5.4 ± 0.5 min) than on the weed species (8 ± 3 min) (*p* < 0.0001, Table 3, Figure 4). The total duration of phloem ingestion was significantly greater for nymphs (202 ± 12) than adults (28 ± 12) (*p* < 0.0001). No differences were observed in the total duration of xylem feeding bouts by psyllids on weeds compared to citrus. (*p* = 0.20). However, adult psyllids (218 ± 18) had longer durations of xylem feeding activities compared to nymphs (113 ± 19) (*p* < 0.0001) during the 18 h of recording (Table 3).

Psyllid life stage had a highly significant effect (*p* = 0.0001) on the total duration (frequency x mean duration) of all detected waveforms (Table 3). Longer total durations of C, D, E1, and E2 waveforms were recorded for nymphs than adults (Table 3). Adults spent more time engaged in G, and Np than nymphs (Table 3). Nymphs engaged in phloem ingestion for durations 7 times longer, on average, than adults (Table 3). Adults spent 1.9 times more time engaged in xylem ingestion than nymphs (Figure 2 and Figure 3, Table 3).

Overall, the total duration of phloem ingestion was significantly longer on citrus plants than on the weed species evaluated. In contrast, no significant differences were observed in the total duration of xylem feeding between the citrus host plant and weed species, which indicates greater generality in xylem than phloem feeding ability among *D. citri* (Table 3). Nymphs spent 60% of their time in phloem ingestion on citrus plants compared to no phloem ingestion on weeds (Figure 2A–D). Adults spent 9% of their time in phloem ingestion on citrus plants and no phloem ingestion (<1%) was observed on weed species (Figure 3A–D). On the other hand, *D. citri* adults (39.5 ± 3.9) performed significantly longer bouts of xylem ingestion than nymphs (21.3 ± 4) (*p* = 0.004) (Table 2). Adults spent 19% to 22% of their total feeding time in xylem ingestion on weeds and citrus plants (Figure 3A–D); whereas, nymphs spent 14% to 15% of their time in xylem feeding activities on weeds and only 3% on citrus plants (Figure 3A–D). In addition, adults spent significantly more time in non-probing (Np) activities on weeds (57%–68%) than on citrus (48%) (Figure 3A–D).

### 3.2. Visualization of Salivary Sheaths Produced by D. citri Adults

The salivary sheaths secreted by groups of *D. citri* adults feeding on the abaxial side of weeds or citrus leaves were examined using fluorescence microscopy of cross sections of the midrib (Figure 4). Fluorescence micrographs showed that *D. citri* adult stylets reached phloem of *C. macrophylla* (Figure 4A), whereas they ended up in the xylem of weed plants such as fennel (Figure 4B), Primrose (Figure 4C) and Spanish needle (Figure 4D). These micrographs are congruent with our EPG data, indicating that *D. citri* generally did not feed on the phloem of weed species. However, adults were successful in reaching the xylem of weed species, which is congruent with successful xylem feeding bouts observed in EPG recordings.

### 3.3. Survival of Psyllid Adults and Nymphs on Weed Species and Citrus in No Choice Assays

**Figure 5:**
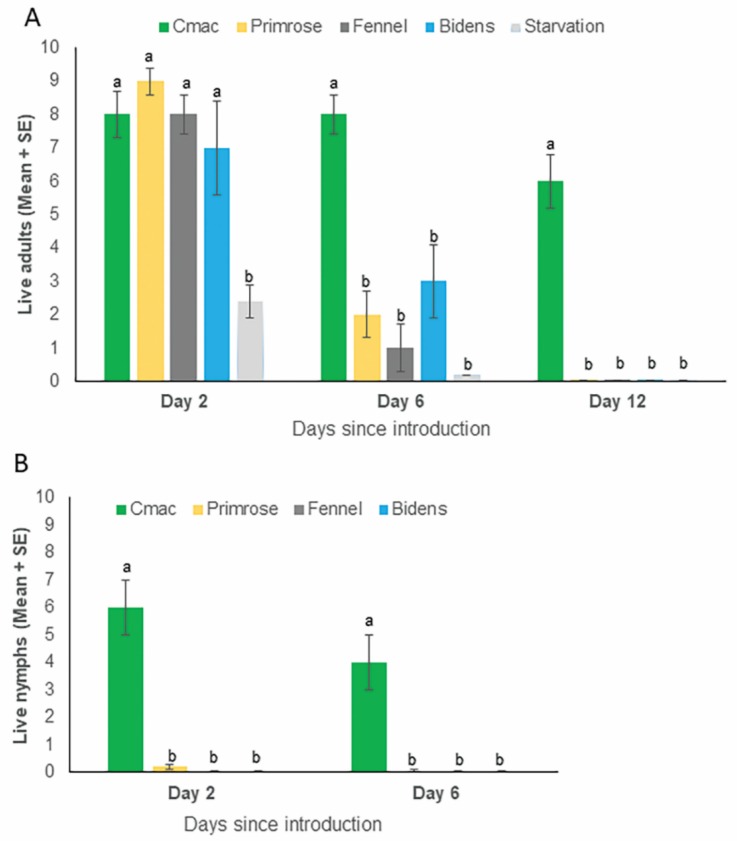
Bar graph showing the survival of *Diaphrina citri* adults (**A**) and nymphal (**B**) instars (means ± SEM) on individual plants of four different plant species (Primrose, Dogfennel, Spanish needle, and *Citrus macrophylla*) and starved control 2, 6, and 12 days after introduction onto plants in clip cages. (**A**) Live adult psyllids on plants and starved control after 2, 6, and 12 days. (**B**) Live fourth instar nymphs on plants after 2 and 6 days. Means analyzed by one-way ANOVA followed by Tukey’s HSD. Treatments having no letters in common are significantly different (*α* = 0.05) (n = 5).

Significantly more adults survived on weed species and *C. macrophylla*, than in the no plant (starvation) negative control (*p* < 0.0001, n = 5) after 48 h (Figure 5A). No differences were observed between survival of adults on weed species and citrus (*p =* 0.67, n = 5) (Figure 5A) after 48 h. After 6 d, the survivorship was highest on *C. macrophylla* (8 ± 0.6) and lowest on the starvation control (0.2 ± 0.2). Adults survived on weed species for up to 6 d (Figure 5A). Each of these weed species tested exhibited an intermediate survivorship between the citrus and the no plant negative control (Figure 5A). However, greater ××mortality of adult psyllids was observed on weed species than on citrus after 6 and 12 d of no choice feeding on these plants (*p <* 0.001, n = 5). No oviposition by *D. citri* adults was observed on any of the weed species evaluated; whereas, oviposition was observed on *C. macrophylla* plants (16 ± 5 eggs/flush). Mortality of nymphs was higher on weed species than on citrus by day 2 of no choice exposure (*p <* 0.001, n = 5). Nymphs were unable to molt into the next instar on weed species for the duration of the assay, whereas 4.3 ± 3.1 nymphs survived on *C. macrophylla* and emerged as adults (Figure 5B).

## 4. Discussion

We investigated the feeding behavior of *D. citri* on weed species that commonly occur in citrus groves to determine if they can act as alternate feeding sources for psyllid nymphs and adults during periods of insecticide sprays and when grove conditions are inhospitable. Although previous research has suggested that these species may act as ‘way stations’ for *D. citri* during dispersal [10], their functional role in the nutritional ecology of this insect was unknown.

Our EPG recordings revealed that *D. citri* nymphs and adults successfully feed on xylem of these weed species and thereby use them as a source of water. The amount of xylem feeding observed on weed species was similar to that observed on preferred citrus host plants. Phloem is more nutrient rich than xylem sap, and is important for nymphal development. Our previous studies showed that adults fed more extensively on xylem than phloem on citrus, compared to nymphs, to maintain water balance [24,25,26]. In the current study, the frequency of xylem feeding bouts on *C*. *macrophylla* was lower than that which occurred on weed species; this type of feeding was otherwise similar in duration per bout and total duration on both non-host weeds and citrus hosts.

Fluorescence micrographs confirmed that adult psyllids accessed the xylem tissues, which allowed extensive bouts of continuous feeding (Figure 4A–D). In contrast, phloem feeding on weed species did not occur in general (only three short phloem-feeding bouts totaling 20 min were observed on primrose) by either nymph or adult psyllids. However, *D. citri* performed multiple unsuccessful attempts to access the phloem tissues, as revealed by phloem penetration (D) and phloem salivation (E1) activities (Table 1). Nymphs performed more phloem ingestion activities (D + E1 + E2) on citrus than adult psyllids, which confirms a previous report and is congruent with observations that nymphs more readily acquire *C*Las than adults [26]. We observed no differences in the frequency of xylem feeding (G) activities between nymphs and adults (Table 1), which indicates that *D. citri* can access the xylem as both immatures and adults. However, the duration of feeding activities was influenced by psyllid life stage. Nymphs performed significantly longer durations of phloem feeding on *C. macrophylla* plants than on weed non-hosts; whereas, adult xylem ingestion was of equivalent length on weeds and citrus. Given that adult psyllids performed minimal or no phloem feeding on weeds, they spent a larger proportion of their overall time performing non-probing activities.

Even though the weed species we chose to investigate here were characterized by softer leaf tissues as compared with new citrus flush growth, the characteristic physical barriers and plant secondary metabolites associated with weeds may have rendered them inferior host plants to *D. citri* by affecting xylem and phloem feeding behaviors. Previously, we showed that the fibrous ring associated with trifoliate citrus, *Poncirus trifoliata* (L.), prevents *D. citri* adults from accessing phloem tissues [24] and contributes to host plant resistance against psyllids that characterizes this citrus genotype [25]. In the current study, psyllids spent the majority of their time performing stylet pathway activities (C), xylem ingestion (G) and non-probing activities on weed species. Multiple phloem penetration and salivation attempts were made on weeds, but none or very few of them resulted in activities characteristic of successful phloem ingestion. Our EPG results indicate that *D. citri* adults may be able to use some of these weed species opportunistically as secondary host plants for acquiring water from xylem tissues, but not as true host plants where nutrition from the phloem could be accessed. Fluorescence micrographs confirmed the behavioral recordings showing that adults successfully penetrated into and ingested from xylem tissues for prolonged durations. Also, some level of salivary sheath branching was observed in the xylem of Spanish needle (Figure 4A), which indicated that psyllids conducted multiple attempts to reach the vascular tissues by actively secreting repeated salivary sheaths. The quality of phloem contents and chemistry of the secondary metabolites of the weed species likely rendered these non-hosts less palatable to *D. citri* relative to preferred hosts.

Weeds can act as alternate sources of food, provide floral nectaries and act as breeding sites for predators and parasitoids, creating favorable microhabitat for beneficial insects [29,30,31,32,33]. Weed management practices may contribute to management of insect vectors that transmit plant pathogens. Colloff et al. [34] reported that citrus monocultures with interspersed weeds suffered less thrips damage than fields without weeds because of higher predator populations. Ortega et al. [35] found that incidence of *D. citri* in the citrus canopy was lower in plots containing an abundance of weeds surrounding trees as compared with trees in plots denuded of weeds. Purposeful growth of weeds among cultivated citrus has been shown to reduce incidence of *D. citri* adults and nymphs in ‘Ortanique’ mandarins [36].

Furthermore, movement of herbivores between crop plants and weeds could support establishment of beneficial insect populations. A good example is the use of refugia or ‘plant banks’ that foster development of beneficial and reduce pest populations. The purposeful cultivation of weeds in citrus may play a beneficial role of improving biological control of *D. citri* populations. However, within the context of the HLB epidemic, the impact of weeds on natural enemy abundance should be weighed against the tradeoff of potentially providing the phytopathogen vector with an alternative refuge where water can be obtained, and lifespan could be extended under harsh conditions. This potential tradeoff as it relates to weed abundance and species composition deserves further investigation.

Occurrence of non-host weeds in or around cultivated citrus may also affect incidence of *D. citri* by interfering with normal host finding using visual cues and/or olfactory cues. Low lying vegetation can influence the spectral reflectance from the ground, affecting the alighting behaviors of flying insects [37]. Conversely, weeds may also modify the attractiveness of crops to insects by affecting perception of foliage color. Hemipteran insects, such as psyllids, use visual cues and are attracted to yellow or greenish yellow color during host-seeking [38,39,40]. Spanish needle and primrose produce yellow flowers and have soft leaf tissue similar to newly emerging citrus leaf flush [10]. It is possible that the spectral reflectance from weeds during unavailability of citrus flushes could influence host-searching behavior of *D. citri*.

## 5. Conclusions

Our EPG recordings and behavioral assays showed that *D. citri* can survive on weed species as a secondary source of xylem in the absence of host plants. Our results provide a feeding mechanism to explain how *D. citri* utilize secondary or alternate hosts for survival following insecticide treatment or during dispersal. Interestingly, our results also showed that both adult and immature *D*. *citri* were generally unable to feed on the phloem of the weed species, limiting their value to the psyllid outside the possibility of their functioning as a short-term refugia.

## Figures and Tables

**Table 1:** Mean (±SEM) frequency (no. of bouts) of feeding activities by *Diaphorina citri* adults and nymphs on weed species and *C*. *macrophylla* during 18 h EPG recordings. Number of replicates: Primrose n = 18, Spanish needle n = 22, Dogfennel n = 18, and *C. macrophylla* n = 22.

		Psyllid Life Stage		Plant Type					
Waveform	Effect	Nymph	Adult	Primrose	Spanish Needle	Dogfennel	*Citrus macrophylla*	*F*-Value	*P* > *F*
C Intercellular passage	Stage	29 ± 2	29 ± 2					0.003	0.95
	Plant type			30 ± 3	32 ± 3	23 ± 3	32 ± 2	3.21	0.02
	Interaction							3.90	0.01
D Phloem penetration	Stage	3.4 ± 0.4	0.7 ± 0.4					21.3	<0.0001
	Plant type			0.09 ± 0.5	0.6 ± 0.5	1.5 ± 0.5	5.1 ± 0.4	21.2	<0.0001
	Interaction							4.6	0.004
E1 Phloem salivation	Stage	3.4 ± 0.4	0.7 ± 0.4					20.6	<0.0001
	Plant type			0.09 ± 0.5	0.6 ± 0.5	1.5 ± 0.5	5.2 ± 0.4	21.4	<0.0001
	Interaction							6.1	0.006
E2 Phloem ingestion	Stage	1.4 ± 0.1	0.3 ± 0.1					22.64	<0.0001
	Plant type			0.08 ± 0.2	0.0 ± 0.0	0.0 ± 0.0	2.8 ± 0.2	58.6	<0.0001
	Interaction							21.9	<0.0001
G Xylem feeding	Stage	6 ± 1	7 ± 1					0.77	0.38
	Plant type			6.9 ± 1	9.6 ± 1	6.8 ± 1	4.3 ± 1	5.1	0.002
	Interaction							2.0	0.11
Np Non-probing activities	Stage	20 ± 1	22 ± 1					0.84	0.36
	Plant type			23 ± 2	22 ± 2	15 ± 2	23 ± 2	3.6	0.01
	Interaction							4.4	0.005

**Table 2:** Mean (±SEM) duration (min) of individual feeding activity bouts by *Diaphorina citri* adults and nymphs on weed species and *C*. *macrophylla* during 18 h EPG recordings. Number of replicates: Primrose n = 18, Spanish needle n = 22, Dogfennel n = 18, and *C. macrophylla* n = 22.

		Psyllid Life Stage		Plant Type					
Waveform	Effect	Nymph	Adult	Primrose	Spanish Needle	Dogfennel	*Citrus macrophylla*	*F*-Value	*P* > *F*
C Intercellular passage	Stage	12.4 ± 0.6	8.9 ± 0.6					16.1	<0.001
	Plant type			9.3 ± 0.9	11.2 ± 0.9	12.3 ± 0.9	9.8 ± 0.8	2.06	0.10
	Interaction							4.4	0.005
D Phloem penetration	Stage	0.8 ± 0.05	0.16 ± 0.05					80.7	<0.0001
	Plant type			0.06 ± 0.07	0.4 ± 0.07	0.5 ± 0.07	0.8 ± 0.06	22.7	<0.0001
	Interaction							6.7	0.0003
E1 Phloem salivation	Stage	0.8 ± 0.1	0.26 ± 0.1					17.6	<0.0001
	Plant type			0.05 ± 0.1	0.3 ± 0.1	0.4 ± 0.1	1.1 ± 0.1	13.6	<0.0001
	Interaction							1.3	0.26
E2 Phloem ingestion	Stage	67.9 ± 10	13.5 ± 10					8.8	0.004
	Plant type			0.2 ± 0.1	0.0 ± 0.0	0.0 ± 0.0	133 ± 13	8.8	<0.0001
	Interaction							23.9	<0.0001
G Xylem feeding	Stage	21.3 ± 4	39.5 ± 3.9					8.5	0.004
	Plant type			37.3 ± 5.8	32.9 ± 5.6	32.3 ± 5.9	22.8 ± 5.1	1.26	0.29
	Interaction							2.09	0.10
Np Non-probing activities	Stage	30 ± 3	41 ± 3					5.10	0.03
	Plant type			41 ± 5	36.4 ± 5	54.6 ± 5	17.2 ± 4	11.85	<0.0001
	Interaction							3.84	0.01

**Table 3:** Mean (±SEM) total duration (frequency x mean duration) (mins) of feeding activities by *Diaphorina citri* adults and nymphs on weed species and *C*. *macrophylla* during 18 h EPG recordings. Number of replicates: Primrose n = 18, Spanish needle n = 22, Dogfennel n = 18, and *C. macrophylla* n = 22.

		Psyllid Life Stage		Plant Type					
Waveform	Effect	Nymph	Adult	Primrose	Spanish Needle	Dogfennel	*Citrus macrophylla*	*F*-Value	*P* > *F*
C Intercellular passage	Stage	316 ± 13	220 ± 12					32.37	<0.0001
	Plant type			239 ± 19	308 ± 18	268 ± 19	252 ± 17	3.40	0.02
	Interaction							8.35	<0.0001
D Phloem penetration	Stage	4 ± 0.5	0.6 ± 0.4					24.6	<0.0001
	Plant type			0.09 ± 0.6	0.7 ± 0.6	1.8 ± 0.6	5.4 ± 0.5	17.1	<0.0001
	Interaction							5.4	0.001
E1 Phloem salivation	Stage	4.3 ± 0.4	0.6 ± 0.4					28.6	<0.0001
	Plant type			0.08 ± 0.6	0.5 ± 0.6	1.6 ± 0.6	6.3 ± 0.5	25.08	<0.0001
	Interaction							10.09	<0.0001
E2 Phloem ingestion	Stage	202 ± 12	28 ± 12					64.1	<0.0001
	Plant type			0.6 ± 0.1	0.00 ± 0.0	0.0 ± 0.0	376 ± 15	133.4	<0.0001
	Interaction							72.1	<0.0001
G Xylem feeding	Stage	113 ± 19	218 ± 18					13.68	0.0003
	Plant type			197 ± 27	188 ± 26	174 ± 28	125 ± 24	1.53	0.20
	Interaction							1.59	0.19
Np Non-probing activities	Stage	437 ± 21	611 ± 20					28.56	<0.0001
	Plant type			642 ± 30	582 ± 29	633 ± 30	315 ± 26	32.74	<0.0001
	Interaction							12.61	<0.0001
